# Beyond the Eye of the Tiger: A Case Report of Adult-Onset Pantothenate Kinase-Associated Neurodegeneration Mimicking Progressive Supranuclear Palsy-Parkinsonism

**DOI:** 10.7759/cureus.115398

**Published:** 2026-08-29

**Authors:** Armon Moradian, Kian Memari, Sergio A Rodriguez, Joren Manuel, Shane Williams, Peter Cohen, Lissette P Lazo

**Affiliations:** 1 Medicine, Dr. Kiran C. Patel College of Osteopathic Medicine, Nova Southeastern University, Fort Lauderdale, USA; 2 Family Medicine, Palmetto General Hospital, Hialeah, USA; 3 Research, Dr. Kiran C. Patel College of Osteopathic Medicine, Nova Southeastern University, Davie, USA; 4 Family Medicine, Dr. Kiran C. Patel College of Osteopathic Medicine, Nova Southeastern University, Fort Lauderdale, USA; 5 Family Medicine, Dr. Kiran C. Patel College of Osteopathic Medicine, Nova Southeastern University, Davie, USA

**Keywords:** basal ganglia iron deposition, dystonia, eye-of-the-tiger sign, nbia, neurodegeneration with brain iron accumulation, pank2, pantothenate kinase–associated neurodegeneration, parkinsonism, pkan, progressive supranuclear palsy

## Abstract

Pantothenate kinase-associated neurodegeneration (PKAN) is an autosomal recessive neurodegeneration with brain iron accumulation disorder that usually begins in childhood or adolescence; very late adult presentation can resemble more common atypical parkinsonian syndromes. We report a 54-year-old woman whose neurologic symptoms began insidiously at approximately 47 years of age with gait and balance impairment, retropulsive falls, micrographia, and dysarthria. Dysphagia, choking on liquids, urinary incontinence, and loss of independence evolved later. She had been labeled as having progressive supranuclear palsy (PSP)-parkinsonism and had no clear sustained response to escalating dopaminergic therapy. During the month before admission, dysphagia and microaspiration accelerated, culminating in acute hypoxic and hypercarbic respiratory failure requiring intubation. Review of outside records showed bilateral globus pallidus and putaminal hypointensity on brain magnetic resonance imaging (MRI), a distribution compatible with neurodegeneration with brain iron accumulation in the appropriate clinical context, and markedly reduced DaTscan uptake in both putamina with reduced right caudate uptake. The available MRI summary did not preserve the exact sequence used to demonstrate basal-ganglia hypointensity and did not document a definite eye-of-the-tiger sign; therefore, low signal was not treated as specific for iron in isolation. Outside molecular testing was documented as identifying pathogenic PANK2 variants, supporting the treating team’s diagnosis of PKAN, although allele-level nomenclature and zygosity were not retained in the available record. Failed extubation led to tracheostomy and percutaneous endoscopic gastrostomy placement. This case illustrates a PSP-like late-adult PKAN phenotype in which chronology, careful interpretation of basal ganglia MRI abnormalities, and molecular testing redirected the diagnosis.

## Introduction

Pantothenate kinase-associated neurodegeneration (PKAN) is an autosomal recessive disorder within the heterogeneous neurodegeneration with brain iron accumulation (NBIA) group. PKAN results from pathogenic variants in PANK2, which encodes mitochondrial pantothenate kinase 2, a key enzyme in coenzyme A biosynthesis. The disorder is characterized clinically by progressive dystonia, dysarthria, rigidity, gait impairment, spasticity, and variably parkinsonism, neuropsychiatric symptoms, or retinal disease, with preferential iron accumulation in the globus pallidus [1,2].

Classic PKAN usually begins in early childhood and progresses rapidly, whereas atypical PKAN has a later onset and slower course, with prominent speech disturbance, psychiatric or behavioral features, dystonia, and parkinsonism [2]. Contemporary cohorts emphasize substantial phenotypic and genotypic heterogeneity; in a 24-patient series, age at onset ranged from infancy to 40 years, and parkinsonism was present in more than half of patients [3]. A presentation beginning near 47 years of age is therefore at the unusually late end of the recognized spectrum.

The clinical overlap with progressive supranuclear palsy (PSP) is important in adult practice. PSP syndromes may include early postural instability and falls, axial akinetic-rigid parkinsonism, dysarthria, dysphagia, and ocular-motor dysfunction; vertical supranuclear gaze palsy or slowed vertical saccades are high-value diagnostic features in the Movement Disorder Society criteria [4]. Atypical PKAN can share falls, rigidity, dysarthria, dysphagia, and poor dopaminergic response, creating a plausible PSP-parkinsonism mimic when the neurogenetic differential is not initially considered.

The classic eye-of-the-tiger sign consists of central T2 hyperintensity surrounded by pallidal hypointensity, but it is neither perfectly sensitive nor entirely specific [5,6]. Hypointensity itself is also not specific for iron and can reflect calcification or other mineral deposition; susceptibility-sensitive sequences and appropriate clinical/genetic correlation strengthen interpretation. Molecular confirmation is therefore especially valuable when imaging is incomplete or atypical. The educational contribution of this case is not a claim of a previously undescribed syndrome, but the combination of unusually late symptom onset, years of PSP-like parkinsonism, severe bulbar progression culminating in respiratory failure, and diagnostic redirection after recognition of an NBIA-compatible imaging pattern and PANK2-associated disease.

## Case presentation

A 54-year-old woman with a chronic neurodegenerative movement disorder previously labeled PSP-parkinsonism presented on August 29 with acute shortness of breath and respiratory distress. The respiratory presentation was abrupt on the day of emergency evaluation but occurred on a background of approximately one month of accelerating dysphagia, inability to swallow safely, intermittent microaspiration while drinking water, and progressive functional decline. She required endotracheal intubation for airway protection and was admitted to the intensive care unit.

Collateral history from her husband and outside neurology records established a longer chronology. Symptoms had begun insidiously at approximately age 47 with progressive gait and balance impairment, recurrent predominantly retropulsive falls, micrographia, and dysarthria. Falls were reported three to four times per month. Dysphagia and frequent choking on liquids became more prominent with time, followed by urinary incontinence and loss of independence in feeding, bathing, and toileting. Her speech was variably intelligible and sometimes rapid. She had received escalating dopaminergic therapy, including a rotigotine patch and extended-release carbidopa-levodopa, without a clear or sustained clinical response; outside records also raised the possibility of levodopa-induced dyskinesias.

Before the index hospitalization, she had been evaluated at outside institutions for the progressive syndrome. The available record notes administration of methylprednisolone 40 mg intravenously during one outside encounter. During the index admission, in the setting of continued diagnostic uncertainty, neurology documented an additional empiric course of methylprednisolone 500 mg intravenously daily for two days while monitoring for clinical change. No sustained steroid-responsive neurologic improvement was documented in the available record. Neurologic examination during the index admission was necessarily limited by intubation and sedation, although the patient could follow commands. Bilateral upper- and lower-extremity rigidity was present. Upward visual tracking was limited, but the available bedside examination did not document a definite supranuclear vertical gaze palsy or clearly slowed vertical saccades. No clear emotional lability was reported in the reviewed outside record. Because the examination occurred during critical illness and sedation, these negative findings were interpreted cautiously rather than as definitive exclusion of PSP. Nevertheless, the absence of a clearly documented core vertical ocular-motor abnormality, together with the broader phenotype and imaging findings, prompted reconsideration of the prior PSP-parkinsonism label.

Outside brain magnetic resonance imaging (MRI) was summarized as showing bilateral globus pallidus and putaminal low signal, mild periventricular T2 fluid-attenuated inversion recovery hyperintensities, and hyperintense signal along the external capsule. The source extract did not preserve whether the basal-ganglia hypointensity was demonstrated on conventional T2-weighted, gradient-recalled echo, or susceptibility-weighted imaging, and no phase or computed tomography correlation was available to independently distinguish iron from calcification or other mineral deposition. Accordingly, the low signal was interpreted as compatible with NBIA in the clinical context rather than as specific evidence of iron by itself [3,5]. The available patient MRI report also did not document a definite central T2 hyperintensity constituting the classic eye-of-the-tiger sign. The prior representative eye-of-the-tiger image has therefore been removed from the revised report to avoid implying that it was the patient's MRI.

The transferred records summarized prior metabolic, infectious, and autoimmune evaluations as unrevealing. However, the assay-level test names, numeric values, and reference intervals were not preserved in the source materials available for this revision; individual negative studies were therefore not reconstructed. Clinically, the differential included PSP-parkinsonism, idiopathic Parkinson disease or another degenerative parkinsonian syndrome, other NBIA disorders, and acquired metabolic, infectious, inflammatory, or toxic causes of dystonia-parkinsonism (Table 1). The unusually late onset, dystonia-parkinsonism with bulbar dysfunction, poor sustained levodopa response, and pallidal/putaminal hypointensity shifted suspicion toward NBIA.

**Table 1:** Differential diagnosis and the reasoning used to reassess the prior PSP-parkinsonism label. NBIA: neurodegeneration with brain iron accumulation; PANK2: pantothenate kinase 2; PKAN: pantothenate kinase-associated neurodegeneration; PSP: progressive supranuclear palsy; MRI: magnetic resonance imaging

Differential	Features supporting consideration	Features favoring PKAN/limitation
Progressive supranuclear palsy-parkinsonism	Retropulsive falls, rigidity, dysarthria/dysphagia, poor levodopa response, limited upward tracking	No definite vertical supranuclear gaze palsy or slowed vertical saccades documented; pallidal/putaminal hypointensity and PANK2 result favored an alternative diagnosis
Idiopathic Parkinson disease	Parkinsonism	Poor sustained levodopa response, prominent dystonia/bulbar disease, early severe postural instability, and MRI pattern were atypical
Other NBIA disorders	Dystonia-parkinsonism and basal-ganglia hypointensity	Remained a radiologic/genetic differential until PANK2-associated disease was documented
Acquired metabolic/mineral disorder	Basal-ganglia low signal can occur with non-iron mineralization	Exact susceptibility/phase imaging and assay-level metabolic testing were not available; low signal was not treated as specific for iron
Inflammatory/infectious/toxic mimic	Progressive movement disorder with prior empiric corticosteroid exposure	Transferred record summarized infectious/autoimmune/metabolic workup as unrevealing; individual assays unavailable

Outside molecular testing was documented in the clinical record as identifying pathogenic variants in PANK2, and the treating neurology team accepted this result as establishing PKAN. The available chart extract did not retain the original laboratory report, Human Genome Variation Society (HGVS) c. and p. nomenclature, zygosity or phase, or the laboratory's American College of Medical Genetics and Genomics/Association for Molecular Pathology (ACMG/AMP) evidence codes. Because those allele-level data could not be verified, the authors did not infer or retrospectively assign specific variants or classifications [7]. This is reported as a source-data limitation rather than supplying unverified molecular details.

During the hospitalization, the patient remained medically fragile because of aspiration-related hypoxic and hypercarbic respiratory failure and severe bulbar dysfunction. A September 1 neurology note documented that she had been extubated the preceding day but required re-intubation early that morning because of increased shortness of breath, increased work of breathing, and inability to clear secretions. On September 2, she underwent percutaneous tracheostomy with bronchoscopic assistance and percutaneous endoscopic gastrostomy placement for durable airway management and enteral nutrition.

Carbidopa-levodopa was restarted in the hospital as a monitored symptomatic trial while neurology reassessed the movement disorder; continuation was not interpreted as evidence that the medication had produced sustained benefit. Baclofen was initiated for rigidity and spasticity, and botulinum toxin remained a consideration for focal dystonia. Rehabilitation, nutritional support, secretion management, and aspiration precautions were incorporated into care. The documented family counseling addressed the progressive nature of PKAN, lack of established curative therapy, and potential clinical-trial eligibility. In view of the autosomal recessive inheritance of PKAN, formal genetic counseling was additionally recommended to review familial-variant confirmation, carrier testing for at-risk relatives, and reproductive options once the molecular report could be obtained [8]. The supplied record does not document completion of cascade testing or reproductive counseling.

The patient's clinical course is summarized in Figure 1, and the diagnostic domains are synthesized in Table 2. At the last available inpatient documentation on September 11, nine days after tracheostomy and gastrostomy placement, she remained markedly rigid, and the neurology team continued PKAN as the diagnosis. No post-discharge clinic note, longer-term respiratory outcome, or subsequent functional assessment was available. A direct patient perspective statement was also not obtainable from the supplied record because communication was limited during critical illness; her husband served as the principal collateral historian and participated in procedural and disease counseling.

**Figure 1:**
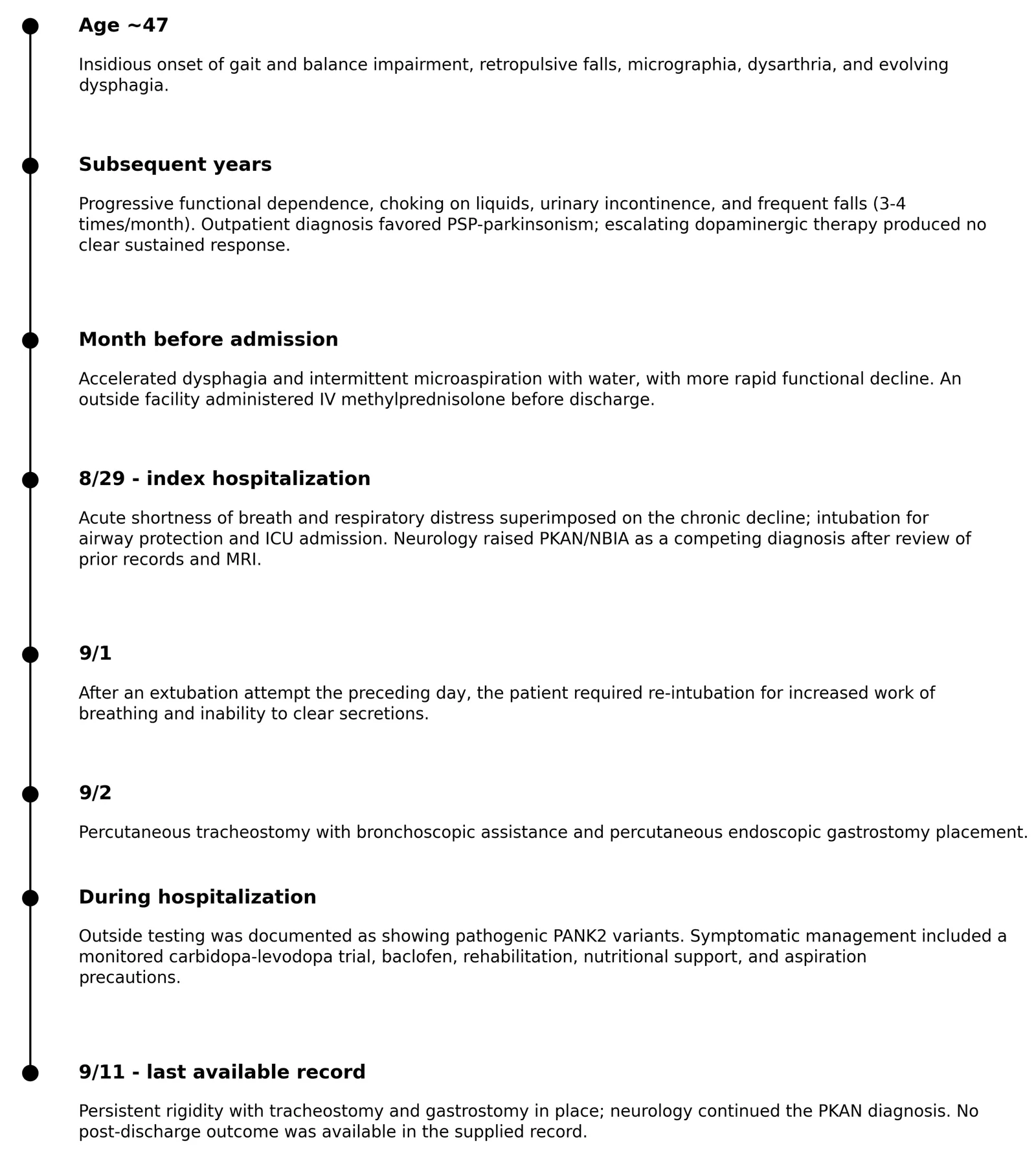
Clinical timeline from symptom onset through the last available inpatient follow-up. Timeline reconstructed from the available clinical record. ICU: intensive care unit; NBIA: neurodegeneration with brain iron accumulation; PANK2: pantothenate kinase 2; PKAN: pantothenate kinase-associated neurodegeneration; PSP: progressive supranuclear palsy; IV: intravenous

**Table 2:** Diagnostic synthesis and chronology of the patient-specific findings. ADL: activities of daily living; ACMG/AMP: American College of Medical Genetics and Genomics/Association for Molecular Pathology; MRI: magnetic resonance imaging; NBIA: neurodegeneration with brain iron accumulation; PANK2: pantothenate kinase 2; PKAN: pantothenate kinase-associated neurodegeneration; PSP: progressive supranuclear palsy; T2-FLAIR: T2-weighted fluid-attenuated inversion recovery; HGVS: Human Genome Variation Society

Symptom onset	Approximately age 47; insidious	Unusually late for PKAN; gait/balance impairment, retropulsive falls, micrographia, dysarthria were early
Later neurologic course	Progressive dysphagia/choking, rigidity, urinary incontinence, ADL dependence	Bulbar and motor progression overlapped with PSP-like parkinsonism
Falls	Retropulsive, reported 3-4 times/month	Compatible with PSP phenotype but not disease-specific
Dopaminergic therapy	Rotigotine and escalating carbidopa-levodopa without clear sustained response	Supported reassessment of the original parkinsonian diagnosis; inpatient use was a monitored symptomatic trial
Ocular-motor examination	Limited upward tracking during intubation/sedation; no definite vertical supranuclear gaze palsy or slowed vertical saccades documented	Core PSP ocular-motor criteria were not established; examination limitation acknowledged
Brain MRI	Bilateral globus pallidus and putamen hypointensity; mild periventricular T2-FLAIR and external-capsule hyperintensity	Distribution compatible with NBIA; exact sequence causing basal-ganglia low signal not retained, so iron was not inferred from low signal alone
Eye-of-the-tiger sign	Not definitively documented in the available patient MRI report	Representative teaching MRI removed to avoid patient-image ambiguity
Molecular testing	Outside record documents pathogenic PANK2 variants	Treating team considered result diagnostic; exact HGVS nomenclature, zygosity/phase, and ACMG/AMP evidence codes unavailable
Acquired-mimic workup	Transferred record states metabolic, infectious, and autoimmune evaluations were unrevealing	Individual assay names and numeric results unavailable; no negative tests were fabricated
Respiratory outcome	Hypoxic/hypercarbic respiratory failure, failed extubation, tracheostomy	Severe bulbar dysfunction and secretion burden drove acute morbidity
Nutritional outcome	Percutaneous endoscopic gastrostomy	Long-term enteral access required because of dysphagia and aspiration risk
Last available follow-up	September 11; persistent rigidity, tracheostomy and gastrostomy in place	No post-discharge or long-term functional outcome available

## Discussion

The principal diagnostic lesson in this case is the degree to which late-onset PKAN can overlap with an adult atypical parkinsonian syndrome. PKAN usually presents far earlier than this patient's onset near 47 years, even within the atypical form [2,3]. Her retropulsive falls, rigidity, dysarthria, dysphagia, poor dopaminergic response, and limited upward tracking created a reasonable initial PSP-like phenotype. At the same time, the bedside record did not establish a definite vertical supranuclear gaze palsy or slowed vertical saccades, which are high-value ocular-motor features in modern PSP criteria [4]. The examination was constrained by sedation and critical illness, so the absence of those findings cannot independently exclude PSP. Instead, the diagnostic pivot arose from integrating the unusual age/course, dystonia-parkinsonism, pallidal/putaminal signal abnormality, and molecular PANK2 result.

Neuroimaging required similar caution. In established PKAN, T2 and susceptibility-weighted MRI commonly show pallidal hypointensity related to iron, and the eye-of-the-tiger sign is frequent but not universal [3,5]. Conversely, an eye-of-the-tiger-like pattern is not absolutely pathognomonic [6]. The patient's outside MRI summary documented pallidal and putaminal hypointensity, but the exact sequence responsible for that finding was not preserved in the available record, and the report did not explicitly describe a classic central T2 hyperintensity. Thus, the imaging should be understood as an NBIA-compatible clue rather than stand-alone proof of iron deposition or PKAN. Removing the previously included representative MRI image avoids conflating a teaching illustration with patient-specific radiology.

Molecular testing is the decisive diagnostic layer when PKAN is suspected. Current diagnostic frameworks describe confirmation by biallelic pathogenic PANK2 variants, and standardized ACMG/AMP criteria provide the framework for laboratory variant classification [7,8]. Recent series continue to expand the PANK2 allelic spectrum and demonstrate the value of segregation analysis and precise variant reporting [3,9]. In this case, the treating record states that pathogenic PANK2 variants were identified, but the original laboratory report was not retained in the materials available to the authors. This prevents reliable reporting of exact HGVS nomenclature, zygosity, phase, or evidence codes and represents an important limitation of the case report. If the original report becomes available, those details should be inserted verbatim rather than reconstructed from phenotype.

The case also illustrates why severe bulbar dysfunction deserves early anticipatory attention in progressive movement disorders. Over several years, dysphagia progressed from choking on liquids to recurrent microaspiration and ultimately hypoxic and hypercarbic respiratory failure. The failed extubation and inability to clear secretions led to tracheostomy and gastrostomy. PKAN treatment remains predominantly supportive and individualized; options include management of dystonia and spasticity, rehabilitation, speech/swallow and nutritional support, respiratory care, and selected procedural therapies. Controlled trials of deferiprone and fosmetpantotenate have not established a broadly effective disease-modifying therapy [10,11], and a recent review continues to characterize treatment as symptom-directed while investigational strategies evolve [12].

Genetic counseling is a clinically meaningful consequence of a PANK2-associated diagnosis. PKAN is autosomal recessive; when both familial pathogenic variants are known, relatives can receive targeted carrier testing, and families can discuss reproductive testing options [8]. The inpatient record documents family counseling about prognosis and clinical trials but does not establish that formal genetics consultation, cascade testing, or reproductive counseling was completed. The revised report therefore distinguishes documented counseling from recommended genetic follow-up.

This report has several limitations. The original molecular laboratory report was unavailable, preventing allele-level reporting. The source MRI extract did not retain the susceptibility sequence or permit independent confirmation of a classic eye-of-the-tiger sign. The chart described metabolic, infectious, and autoimmune investigations as unrevealing but did not retain the individual assay results used to exclude acquired mimics. Neurologic examination was limited by intubation and sedation, and follow-up ended with inpatient documentation on September 11. These constraints limit phenotypic precision and prevent conclusions regarding long-term outcome, but they also underscore the importance of preserving original imaging and molecular reports when reporting rare neurogenetic disease.

## Conclusions

This case adds a clinically instructive example of PKAN beginning unusually late in adulthood and initially resembling PSP-parkinsonism. The diagnostic red flags were the prolonged dystonia-parkinsonism course with recurrent retropulsive falls and severe bulbar dysfunction, lack of sustained dopaminergic benefit, basal-ganglia hypointensity compatible with an NBIA disorder, and a documented pathogenic PANK2 molecular result despite the absence of a clearly documented classic eye-of-the-tiger sign.

For adults with PSP-like parkinsonism and atypical basal-ganglia MRI abnormalities, careful chronology and molecular testing can clarify the diagnosis when bedside features are incomplete or overlapping. In this patient, the immediate outcome was stabilization with tracheostomy and gastrostomy after aspiration-related respiratory failure; the available record ended on September 11 and does not support claims about longer-term neurologic or functional improvement.
